# Incidence and prevalence of upper-extremity musculoskeletal disorders. A systematic appraisal of the literature

**DOI:** 10.1186/1471-2474-7-7

**Published:** 2006-01-31

**Authors:** Bionka MA Huisstede, Sita MA Bierma-Zeinstra, Bart W Koes, Jan AN Verhaar

**Affiliations:** 1 Erasmus MC, Department of General Practice, Rotterdam, The Netherlands; 2 Erasmus MC, Department of Orthopaedic Surgery, Rotterdam, The Netherlands

## Abstract

**Background:**

A systematic appraisal of the worldwide incidence and prevalence rates of UEDs available in scientific literature was executed to gauge the range of these estimates in various countries and to determine whether the rates are increasing in time.

**Methods:**

Studies that recruited at least 500 people, collected data by using questionnaires, interviews and/or physical examinations, and reported incidence or prevalence rates of the whole upper-extremity including neck, were included.

**Results:**

No studies were found with regard to the incidence of UEDs and 13 studies that reported prevalence rates of UEDs were included. The point prevalence ranged from 1.6–53%; the 12-months prevalence ranged from 2.3–41%. One study reported on the lifetime prevalence (29%). We did not find evidence of a clear increasing or decreasing pattern over time. The case definitions for UEDs used in the studies, differed enormously. Therefore, it was not possible to pool the data.

**Conclusion:**

There are substantial differences in reported prevalence rates on UEDs. Main reason for this is the absence of a universally accepted way of labelling or defining UEDs. If we want to make progress in this field, the first requirement is to agree on unambiguous terminology and classification of EUDs.

## Background

Upper extremity disorders (UEDs) are a major problem in modern society. Besides the impact on patients themselves, the disorders also form a huge economic burden due to costs for sick leave and health care. UEDs affect people all over the world. In the early 1980's in Australia Hocking [1] even reported an epidemic disease of a disorder he called RSI (repetitive strain injury). Numerous other terms have been used to indicate UEDs such as cumulative trauma disorders, physical overuse syndrome, and occupational cervicobrachial disorders. UEDs comprise various clinically defined (e.g. carpal tunnel syndrome) and undefined conditions of muscles, tendons, or nerves in the upper extremity due to multiple factors. Not only occupational use of the upper limbs, but also psychosocial work characteristics such as high job stress [2], high job demand [3], non-work-related stress [2] and personal characteristics such as coping [4] can cause UEDs. Most UEDs are manifested by pain, discomfort, or tingling in the upper extremity [5].

In medical literature authors repeatedly suggested that during the last decade's data are reported to indicate the extent, and in some cases increase of UEDs over time in Australia, Canada, the USA, France, The Netherlands, and elsewhere [1,2,6-11]. For example, based on workers' compensation claims Silverstein et al [12] reported a dramatic increase of UEDs since the early 1980s in the USA affecting workers in virtually every industry. In 1981, 28,6% of the allowed workers' compensation claims in New York State concerned UEDs and by 1986, these numbers were increased by 10,2% [13]. In 1989 the total U.S. workers compensation costs for UEDs was estimated to be $563 million [14]. Also in the early 1990s UEDs have dramatically increased in incidence according the data from the U.S. Bureau of Labor Statistics, 1998a [15].

In Ontario, Canada, UEDs constituted up to 24% of lost-time workers compensation claims in 1992 [16]. In 2000/01, one in ten Canadians aged 20 or older reported an UED that was serious enough to limit their normal activities in the previous 12 months [17]. In 2000 the Health Council of the Netherlands reported that if no distinction is made on the basis of duration or seriousness, the prevalence of UEDs in the Netherlands was between 20 and 40 percent [18].

A systematic appraisal of worldwide incidence and prevalence studies may permit us to gauge the range of incidence and prevalence of UEDs in various countries and, where possible, to pool data. It provides the basis for determining whether these estimates of UEDs are increasing over time. The data are also needed to estimate the size of study populations for experimental and preventive trials. Therefore, a systematic appraisal of the worldwide incidence and prevalence rates of UEDs reported in available studies will be presented here.

## Methods

### Literature search

Studies were identified by searches of the computerized bibliography database Medline (1966 to June 2004). All the keywords mentioned for UEDs in relevant articles were used in the literature search, such as repetitive strain injury (RSI), upper-extremity disorders (UED), work-related musculoskeletal disorders (WMSD), and cumulative trauma disorders (CTD). In order to identify relevant studies for this review, these keywords were combined with the terms "prevalence" and "incidence" in the title or abstract. On the basis of title and abstract articles were excluded in which prevalence and UEDs were no issue. Full texts of the remaining articles were assessed on eligibility.

### Eligibility of studies

Studies were eligible for inclusion if (1) at least 500 people were included in the study; (2) incidence or prevalence rates of UEDs were reported for the whole upper-extremity region including neck and (3) data were collected by using questionnaires, interviews and/or physical examinations. When incidence or prevalence rates were only presented for neck, shoulder, elbow or hand separately, the study was excluded. Studies based on administrative data such as data from workers' compensation claims or from registrations of occupational health services were excluded because these studies may represent changes in administrative policy and economical matters rather than the actual incidence or prevalence.

Studies that recruited persons from the open population as well as from a selected population (working, non-working, primary care, secondary care, etc.) were included. Only studies written in English, French, German, and Dutch were considered.

### Data extraction

Relevant data were collected from eligible studies on standardized forms concerning incidence or prevalence rates, the used term, definition for UEDs, the year of measurement, the setting and the country in which the study was carried out.

### Pooling of data

Before data can be pooled from different studies, the homogeneity of the data should be taken into account. The minimum criteria for data pooling in this systematic appraisal were the use of similar case definitions of UEDs, homogeneity of the study population, and the use of similar types of incidence or prevalence rates.

## Results

### Study selection

The search strategy resulted in a total of 523 studies. After the first eligibility screening, based on title and abstract, 206 potentially relevant articles were identified. Reviewing full text articles, 47 studies reporting incidence or prevalence of UEDs consisting of a population of 500 cases or more were found. Of these, 13 studies met the inclusion criteria. They all reported prevalence rates. No studies were found with regard to the incidence of UEDs that met the inclusion criteria. One study [19] was found that studied the prevalence in nurses by asking the following question: "Do you suffer regularly from arm or neck complaints?" Because 'regularly' is not defined we decided to exclude this study. The other 33 studies were excluded for the following reasons: they reported incidence rates instead of prevalence rates; they did not report prevalence rates of the whole upper extremity, etc.

### Study characteristics

The 13 studies included in this review are presented in table 1, together with their relevant characteristics. All studies were published between 1987–2003; the data of the studies were collected between 1983–1998. Six studies were executed in the USA. In Canada two studies were carried out. The other studies were from Australia, England, Italy, The Netherlands and Sweden. The majority of the studies (seven) focused on a working population that was expected to be at high risk for UEDs, whereas two studies focused on a low risk working population. Two studies concerned students and the other two studies were carried out in the general population.

The studies reported different types of prevalence rates, i.e., point prevalence (six studies), 12-month prevalence (six studies) and lifetime prevalence (one study). The occurrence of UEDs was assessed either through questionnaires (eight studies), a telephone interview (one study), a questionnaire and clinical examination (two studies), or an interview and a clinical examination (two studies).

### Case definition of UEDs

A diversity of terms and case definitions for UEDs were used (table 2). Three of the six studies reporting point prevalence rates [20-22] did not present any definition of UEDs. Ehrmann Feldman et al [23] defined UEDs as 'having substantial neck and upper limb pain' and Fry [24] described the 'overuse (injury) syndrome' as 'those changes brought about in the muscle and joint ligaments from excessive use, causing pain, loss of function, and almost always demonstrable tenderness in the affected structure'. McCormack et al [25] used in addition a (specified) physical examination to define UEDs.

One of the six studies reporting 12-months prevalence rates did not give a definition of UEDs. The authors of this study [26] reported about neck and upper extremity symptoms without any specification. Hales et al [27] defined cases of UEDs using symptom questionnaire and physical examination. Morse et al [28] defined UEDs as 'pain or discomfort of the hand, arm, shoulder, or neck for one continuous week or 20 days total over the previous 12 months (= chronic pain)'. The other three studies [16,29,30] also reported a specified definition of UEDs. The terms they used refer to musculoskeletal disorders located in the upper extremity. In the case definitions they specified the duration of the complaints within the last 12 months and the sensation the patients must have beside pain such as discomfort or paraesthesia. In addition, Batavi et al [30] and Bernard et al [29] excluded UEDs caused by an acute trauma. Bernard et al [29] made the case definition even more specific by labeling work-relatedness of the disorder caused by the current job and the seriousness of the disorder.

Stockstill et al [31], reporting lifetime prevalence, used the term 'upper extremity neuropathy' and defined the conditions as 'altered sensation in hands or arms, forearms, cervical area or neck'.

### Pooling data

The first requirement to enable pooling of data is the use of similar case definitions of UEDs across studies. The case definitions used in the 13 included studies, as illustrated above, differed enormously. None of the studies used the same or a similar description of UEDs. Therefore, it is not possible to pool the data and the results will be described.

### Prevalence rates of UEDs

#### Point prevalence

Point prevalence ranged from 1.6–53.0%. The point prevalence rates of self-reported complaints in the working population and students were higher (30.0–53.0%) then the point prevalence rates acquired by physical examinations (range 9.3–26.9). The highest point prevalence rates were reported in the USA within textile workers and students, 47% and 53% respectively [25,32], although McCormack et al [25] reported a lower point prevalence rate of 26.9% after physical examination of the positive cases according the results of the questionnaire. In the Dutch open population the lowest point prevalence rates were measured. The rate in male and females being similar (2%) [22]. A prevalence rate of 30% was reported in the late 1990's in people with non-manual occupations in England [21].

#### 12-months prevalence

The 12-months prevalence ranged from 2.3–41,0%. Dimberg et al [26] reported a 12-months prevalence rate (23%) of self-reported complaints in the early 1980s in aircraft engineers, consisting of 86% males. The 12-months prevalence rate of self-reported complaints in newspaper employees in the early 1990s in the USA and Canada was 41% and 19.8%, respectively [16,29]. The study population of the newspaper employees in the USA included more females than the study population in Canada (59% and 44%, respectively). Morse et al [28] reported a 12-months prevalence rate of 11.7% in workers in Connecticut (USA), in 1996. The 12-months prevalence rate of complaints collected by using a questionnaire and a physical examination in high-risk telecommunication employees in the USA was 22% [27]. In a population in Italy that is not occupational exposed to tasks implying repetitive and/or forced movements of the upper limbs Batevi et al [30] reported that the 'anamnestic cases' of UEDs occurred in about 2% of persons aged 15 to 35 years; in persons aged 35 years and older the prevalence rate increased to more than 7%. After clinical examination of the positive anamnestic cases, however, the prevalence rates of both age groups decreased to 0.5% and 3.4% respectively.

#### Lifetime prevalence

In just one study [31] the lifetime prevalence was estimated. In this study in dentists the lifetime prevalence was estimated to be 29%.

## Discussion

In this systematic appraisal worldwide incidence and prevalence rates for UEDs available in scientific literature were collected. No studies were found with regard to the incidence of UEDs that met the inclusion criteria. The estimates of the prevalence rates varied enormously across the 13 included studies. The point prevalence ranged from 1.6–53% and the 12-months prevalence ranged from 2.3–41%. One study reported on the lifetime prevalence (29%). Only Picavet et al [22] studied the prevalence in an open population. The low point prevalence they reported can not be compared with the other studies available, because they all studied a specific (working) population. In addition, Picavet et al [22] reported on the occurrence of 'RSI', while the occurrence of an epicondylitis (around 11%) and a tendonitis or capsulitis (for the whole body they reported a prevalence rate around 16%) were reported separately and therefore not included in 'RSI'.

In this study studies were included that reported incidence and prevalence rates of the whole upper extremity. Studies, which reported incidence or prevalence rates on different regions of the upper extremity separately, but give no estimates for the whole upper extremity, were excluded. Reviews on the prevalence rates of a specific disorder or complaints in one region of the upper extremity have been reported elsewhere. For example, the estimates of the occurrence of the Carpal Tunnel Syndrome in different occupational groups was studied by Hagbert et al [33] and varied between 0.6 and 61%. Luime et al [34] reported on prevalence rates of shoulder pain studied in open population: the point-prevalence ranged from 7 – 27% and the 12-months prevalence ranged from 8.4 – 20%

In general, higher prevalence rates of UEDs were found in women then in men and the estimates of self-reported complaints were higher than those acquired by using (in addition) physical examinations. No evidence of a clear increasing or decreasing pattern over time was found. Although period prevalence can be more biased then point prevalence because of incomplete response or due to recall bias [35], 'firm' conclusions can not been drawn because of the diversity of terms and definitions of UEDs used in the included studies.

To describe the conditions a variation of terms such as 'pain', 'disorders', 'complaints', 'syndrome', 'symptoms', and 'injury' are used in the literature. Because of the different meanings of the terms, it is important to give sound arguments when using certain terms. For example, it you want to describe specific and non-specific cases, using the term disorder is not very clear, because a 'disorder' indicates a specific disease, which can be diagnosed by fixed criteria. All terms used for UEDs in the included studies, except those used by Picavet et al [22] and Fry et al [24] indicated the location of the condition. In our opinion, it is practical and functional to use the localization of the conditions in the term.

Although the term used for UEDs is important because of the perception it causes and the clarity of the medical condition, the definition is even more important. This is not only the case for researchers when they want to compare data of different studies, but also for medical and paramedical staff, so they can speak in an unambiguous way or 'language'. This unambiguous 'language' has to make sure that physicians and other healthcare workers have in mind and speak about the same condition when they discuss the subject or, for example to evaluate the (multidisciplinary) treatment of one of their patients. The case definitions used in the included studies varied enormously. Although studies reporting prevalence rates for UEDs were not included and this appraisal was limited to studies which included 500 cases or more and studies of which the data were published in scientific literature, the diversity of case definitions and classification of UEDs that was found was substantial. This is a general problem and reported in literature by many authors before [36-39].

The diversity in terms and case definitions of EUDs in the included 13 studies prevented any meaningful pooling of data. Drawing comparisons between countries, different working population and assessment of changes in time within a population or country could therefore not be carried out in a quantitative manner.

Different questionnaires and tests used for the physical examinations were presented in the studies; little was said about the validity and reliability of the measurement tools. Developing criteria for classification or diagnosis would be easy if gold-standard diagnostic tests would be available. Unfortunately, no criterion standard for any of the upper extremity soft tissue musculoskeletal conditions is available [37].

If we want to make progress in this field, the first requirement is to agree on unambiguous terminology and classification of EUDs. Physicians and other healthcare workers dealing with patients with these conditions should be involved in such a project. Studies of classification criteria suggest that expert clinicians can more accurately identify cases than most history, physical examination, or laboratory parameters [40]. Furthermore, involving all key disciplines dealing with patients with UEDs will make implementation of the results more successful. Therefore, a multidisciplinary project on national or international level in which all key disciplines cooperate with the intention to achieve multidisciplinary consensus on terminology and classification of UEDs is recommended. When they have agreed about an 'unambiguous' language, the next step is to achieve consensus about valid diagnostic criteria for UEDs and to study the best (multidisciplinary) prevention and/or treatment.

## Conclusion

No studies were found with regard to incidence rates of UEDs and there are substantial differences in reported prevalence rates on UEDs. One of the main reasons for this is the absence of a universally accepted way of labelling or defining UEDs. Health professionals and policy makers should be aware of this problem when they estimate the occurrence of the conditions in populations and the necessary demand and related costs for health care.

## Competing interests

The author(s) declare that they have no competing interests.

## Authors' contributions

BMAH and SMAB have made substantial contributions to conception and design. BMAH did the literature search, data extraction and drafted the manuscript. SMAB, BWK and JANV have helped interpreting the studies found to include in the review and critically revised the manuscript. All authors read and approved the final manuscript.

## Pre-publication history

The pre-publication history for this paper can be accessed here:



## Figures and Tables

**Table 1:** Characteristics of the study populations

*Study*	*Year of data collection*	*Country*	*Study population*	*Number studied*	*Response rate*	*Age (years)*	*Females*
**Studies reporting point prevalence**							
Fry et al. 1987	1985	Australia	Music population (7 performing music schools)	1249	-	-	55%
McCormack et al. 1990	-	USA	Textile workers	1) 2047 2) 895	91% 94%	33.0–38.1 -	75.8% -
Feldman et al. 2002	1995	Canada	High school students	502	62%	13.8 (0.1)	47.4%
Palmer et al. 2001	1997–98	Great Britain	Non-manual occupations	4889	58%	16–64	53.4%
Picavet et al. 2003	1998	The Netherlands	Open population	3664	46.9%	>25	49.6%
Katz et al. 2000	1998	USA	College students	1544	96%	-	45.5%
**Studies reporting 12-months prevalence**							
Dimberg et al. 1989	1983	Sweden	Engineering industry	2814	96%	>10	13.6%
Bernard et al. 1994	-	USA	Newspaper employees	973	93%	39.2 (10.5)	59.3%
Hales et al. 1994	-	USA	Tele- communication employees	518	-	37.5 (9.8)	-
Polanyi et al. 1997	1995	Canada	Newspaper employees	1007	84%	42 (9.4)	44%
Batevi et al. 1998	-	Italy	Kindergarten teachers and traffic policemen	749	-	15–35 (42%) >35 (58%)	15–35 (55.9%) >35 (60.1%)
Morse et al. 2003	1996	USA	Connecticut workers	3200	78%	Working age	-
**Studies reporting lifetime prevalence**							
Stockstill et al. 1993	1991	USA	Dentists	1016	98%	-	-

**Table 2:** Overview of terminology and prevalence rates

*Country, year of data collection (reference)*	*Term*	*Definition*	*Measurement tool*	*Prevalence (%)*		
				
				*Total*	*Men*	*Women*
**Point prevalence**						
Australia, 1985 (Fry et al. 1987)	Overuse (injury) syndrome	Those changes brought about in the muscles and joint ligaments form excessive use, causing pain, loss of function, and almost always demonstrable tenderness in the affected structures	Interview and examination (most) of the effected cases	9.3	3.2	6.1
USA, – (McCormack et al. 1990)	Upper extremity disorders	1) Current problems in the upper extremity 2) ICD.9 CM used to code diagnosis. Excluded osteoarthritis, previous trauma unrelated to present employment, and rheumatic diseases	1) Questionnaire 2) Physical examination of the positive cases	47 26.9	- -	- -
Canada, 1995 (Feldman et al. 2002)	Neck and upper limb pain	Having substantial neck and upper limb pain at inception	Questionnaire	31.9	-	-
Great Britain, 1997–98 (Palmer et al. 2001)	Upper limb symptoms (inclusive neck)	-	Questionnaire	30.0	-	-
The Netherlands, 1998 (Picavet et al. 2003)	RSI Repetitive strain injury	-	Questionnaire	1,9	2.0	1.9
USA, 1998 (Katz et al. 2000)	Upper extremity musculo- skeletal disorders	-	Questionnaire	53.0	21.2	31.8
**12-months prevalence**						
Sweden, 1983 (Dimberg et al. 1989)	NES Neck and upper extremity symptoms	-	Questionnaire	23.0	-	-
USA, 1991 (Bernard et al. 1994)	Musculo- skeletal disorders of the upper extremity	Pain, numbness, tingling, aching, stiffness, or burning in neck, shoulder, hand, or wrist and all of the following criteria applied: 1) no previous accident or sudden injury that was work-related 2) symptoms began after the current job was started 3) symptoms lasted for more than one week or occurred at least once a month within the last year 4) symptoms were reported as "moderate" or "worse" of a five-point intensity scale.	Questionnaire	41.0	-	-
USA, – (Hales et al. 1994)	Work-related UE disorders Upper extremity disorders	Pain, aching, stiffness, burning, tingling, or numbness Symptoms occurred within the past year No previous accident or trauma within the past year Symptoms began after employment within the company Symptoms occurred on the current job Symptoms lasted for more than 1 week, or occurred at least once a month Positive findings on the symptomatic joint area (criteria defined for various medical conditions)	Questionnaire and physical examination	22.0	2.8	17.2
Canada, 1995 (Polanyi et al. 1997)	Upper limb (neck, shoulder and arm) work-related musculo- skeletal disorders (WMSDs)	Those who experienced moderate, severe, or unbearable pain or discomfort either once per month or for longer than a week over the past year	Questionnaire	19.8	7.7	12.1
Italy, – (Batevi et al. 1998)	WMSDs work-related musculo- skeletal disorders of the upper limbs	'Anamnestic cases': pain or paraesthesia present for at least 1 week during the previous 12 months, or appearing at least once a month, and not subsequent to acute trauma	Anamnestic	1.9 age 15–35 7.2 age >35 9.1 total age >15	0.8 age 15–35 2.7 age >35 3.5 total age >15	1.1 age 15–35 4.5 age >35 5.6 total age >15
		'Clinical examination' (cases out of the positive anamnestic cases): no definition	Clinical examinations	0.5 age 15–35 3.4 age >35 3.9 total age >15	no data age 15–35 0.8 age >35 0.8 total age >15	0.5 age >35 2.6 age >35 3.1 total age >15
USA, 1996 (Morse et al. 2003)	UEMSD Upper extremity musculo- skeletal disorders	Pain or discomfort of the hand, arm, shoulder, or neck for one continuous week or 20 days total over the previous 12 months (= chronic pain)	Telephone survey	11.7	-	-
**Lifetime prevalence**						
USA, 1991 (Stockstill et al. 1993)	Upper extremity neuro- pathy	Altered sensation in hands or arms, forearms, cervical area or neck	Questionnaire	29	-	-
